# ACKR3 Antagonism Enhances the Repair of Demyelinated Lesions Through Both Immunomodulatory and Remyelinating Effects

**DOI:** 10.1007/s11064-024-04173-1

**Published:** 2024-05-31

**Authors:** Laetitia Pouzol, Anna Sassi, Mélanie Tunis, Anaïs Zurbach, Nadège Baumlin, Carmela Gnerre, Daniel S. Strasser, Julia Marrie, Enrico Vezzali, Marianne M. Martinic

**Affiliations:** Idorsia Pharmaceuticals Ltd, Hegenheimermattweg 91, Allschwil 4123, Basel-Landschaft, Switzerland

**Keywords:** ACKR3/CXCR7, CXCL12, CXCL11, Multiple sclerosis, Remyelination, Immunomodulation

## Abstract

Addressing inflammation, demyelination, and associated neurodegeneration in inflammatory demyelinating diseases like multiple sclerosis (MS) remains challenging. ACT-1004-1239, a first-in-class and potent ACKR3 antagonist, currently undergoing clinical development, showed promise in preclinical MS models, reducing neuroinflammation and demyelination. However, its effectiveness in treating established disease and impact on remyelination after the occurrence of demyelinated lesions remain unexplored. This study assessed the therapeutic effect of ACT-1004-1239 in two demyelinating disease models. In the proteolipid protein (PLP)-induced experimental autoimmune encephalomyelitis (EAE) model, ACT-1004-1239 administered upon the detection of the first signs of paralysis, resulted in a dose-dependent reduction in EAE disease severity, concomitant with diminished immune cell infiltrates in the CNS and reduced demyelination. Notably, efficacy correlated with elevated plasma concentrations of CXCL11 and CXCL12, two pharmacodynamic biomarkers of ACKR3 antagonism. Combining ACT-1004-1239 with siponimod, an approved immunomodulatory treatment for MS, synergistically reduced EAE severity. In the cuprizone-induced demyelination model, ACT-1004-1239 administered after 5 weeks of cuprizone exposure, significantly accelerated remyelination, already quantifiable one week after cuprizone withdrawal. Additionally, ACT-1004-1239 penetrated the CNS, elevating brain CXCL12 concentrations. These results demonstrate that ACKR3 antagonism significantly reduces the severity of experimental demyelinating diseases, even when treatment is initiated therapeutically, after the occurrence of lesions. It confirms the dual mode of action of ACT-1004-1239, exhibiting both immunomodulatory effects by reducing neuroinflammation and promyelinating effects by accelerating myelin repair. The results further strengthen the rationale for evaluating ACT-1004-1239 in clinical trials for patients with demyelinating diseases.

**Supplementary Information:**

The online version contains supplementary material available at 10.1007/s11064-024-04173-1.

## Background

Multiple sclerosis (MS) is a chronic immune-mediated demyelinating disease of the central nervous system (CNS), considered as one of the most common causes for disability in young adults. The most prevalent presentation of MS at onset is a relapsing-remitting (RR) course, characterized by periods of neurological deficits followed by complete or incomplete recovery in patients. However, most patients diagnosed with RRMS later transition to a progressive disease form, marked by slow expanding lesions characterized by activated macrophages and microglia at the lesion edge, failure in remyelination, and progressive axonal loss [1, 2]. The mechanisms underlying the transition from a disease state where myelin sheath repair is possible to a form marked by the accumulation of lesions and subsequent development of long-term disability, remain poorly understood. Current approved disease modifying therapies, which mainly target peripheral immune cell activation and their entry into the CNS, do not completely halt nor reverse the progression of the disease. Thus, therapeutic strategies that can target both neuroinflammation and remyelination could be beneficial to promote CNS repair and prevent disease progression over drugs with only anti-inflammatory/immunomodulatory effects [3]. ACKR3, an atypical chemokine receptor formerly known as CXCR7, has been proposed as a potential therapeutic target to simultaneously reduce neuroinflammation and promote remyelination [4–6]. ACKR3 is constitutively expressed and is upregulated throughout the CNS during demyelination in several nonclinical models of MS, especially in meningeal and parenchymal blood vessels, on astrocytes, and on Olig2-expressing oligodendrocyte precursor cells (OPCs) [7–9]. Several studies point to a pathological role of the ACKR3 axis during neuroinflammatory demyelinating diseases, specifically (a) promotion of immune cells infiltration into the CNS [4], (b) contribution to chemotaxis and phagocytosis of macrophages/microglia [10–12], (c) modulation of astrocyte functions [9, 13, 14], and (d) contribution to demyelination processes [8]. While some of these pathological effects have been proposed to be linked to the potential signaling capacity of ACKR3, most of them are believed to result from the scavenging activity of ACKR3 that reduces local extracellular levels of its chemokine ligand, CXCL12 [7, 8]. In line with these data, blocking ACKR3 with ACT-1004-1239, a potent and selective ACKR3 antagonist, increased plasma CXCL12 concentrations both in mice and humans [15]. This dose-dependent increase in plasma CXCL12 upon ACKR3 blockade correlated with reduced disease severity in an experimental autoimmune encephalomyelitis (EAE) model, an auto-immune model of MS [6]. In addition, in line with previous studies using a functional ACKR3 antagonist [8], treatment with ACT-1004-1239 was also able to reduce the demyelination induced by the toxic agent cuprizone in mice, emphasizing the pathological role of ACKR3 in preclinical models of demyelinating diseases [6]. However, therapeutic efficacy of ACKR3 antagonism on established EAE disease or on remyelination after initial exposure to cuprizone has not been studied yet. In addition, the strain of mice used in previous efficacy studies does not express the second chemokine ligand of ACKR3, namely the IFN-γ inducible chemokine CXCL11 [16], which also binds to CXCR3. As such, the immunomodulatory effect of ACT-1004-1239 in EAE and its potential indirect impact on the CXCL11/CXCR3 axis has not previously been investigated.

To elucidate whether ACKR3 antagonism is efficacious on established neuroinflammatory demyelinating diseases in a mouse strain that expresses both ligands for ACKR3, ACT-1004-1239 was first evaluated, in a therapeutic setting, in the proteolipid protein (PLP)-induced EAE model in SJL mice. In addition, to investigate the effects of ACKR3 blockade on remyelination, ACT-1004-1239 was administered following exposure to the demyelinating agent cuprizone.

## Materials & Methods

### Mice

Female SJL/J and male C57BL/6 mice were purchased from Charles River Laboratories (France). All mice were housed in groups in a light- and climate-controlled environment and allowed to acclimatize for at least 7 days before use. Mice had free access to food and drinking water *ad libitum* and were used at 8–10 weeks of age after acclimation. All animal experiments were carried out in accordance with the Swiss animal protection law, under protocols approved by the Basel Cantonal Veterinary Office, Switzerland.

### Test Compounds

The ACKR3 antagonist ACT-1004-1239 was synthesized as previously described [17]. Siponimod and Clemastine fumarate were purchased from MolCore Biopharmatech Co, Ltd (Hangzhou, China) and from Toronto Research Chemicals (Toronto, Canada), respectively. For in vivo studies, ACT-1004-1239, siponimod, and clemastine fumarate were all formulated in 0.5% methylcellulose (Sigma-Aldrich, Schnelldorf, Germany) and 0.5% Tween 80 (Sigma-Aldrich) in water and administrated orally (p.o.) in a volume of 5 ml/kg/administration at doses and time indicated in the figure legends. ACT-1004-1239, being a high-clearance drug in rodents [17], was administered twice a day (b.i.d.) with a night window of maximum 14 h (trough concentration). To achieve similar stress levels across all treatment groups, mice administered siponimod or clemastine once daily (q.d.) also underwent an additional oral gavage with the vehicle.

### Proteolipid Protein Peptide (Plp)-Induced EAE Model

**Fig. 1:**
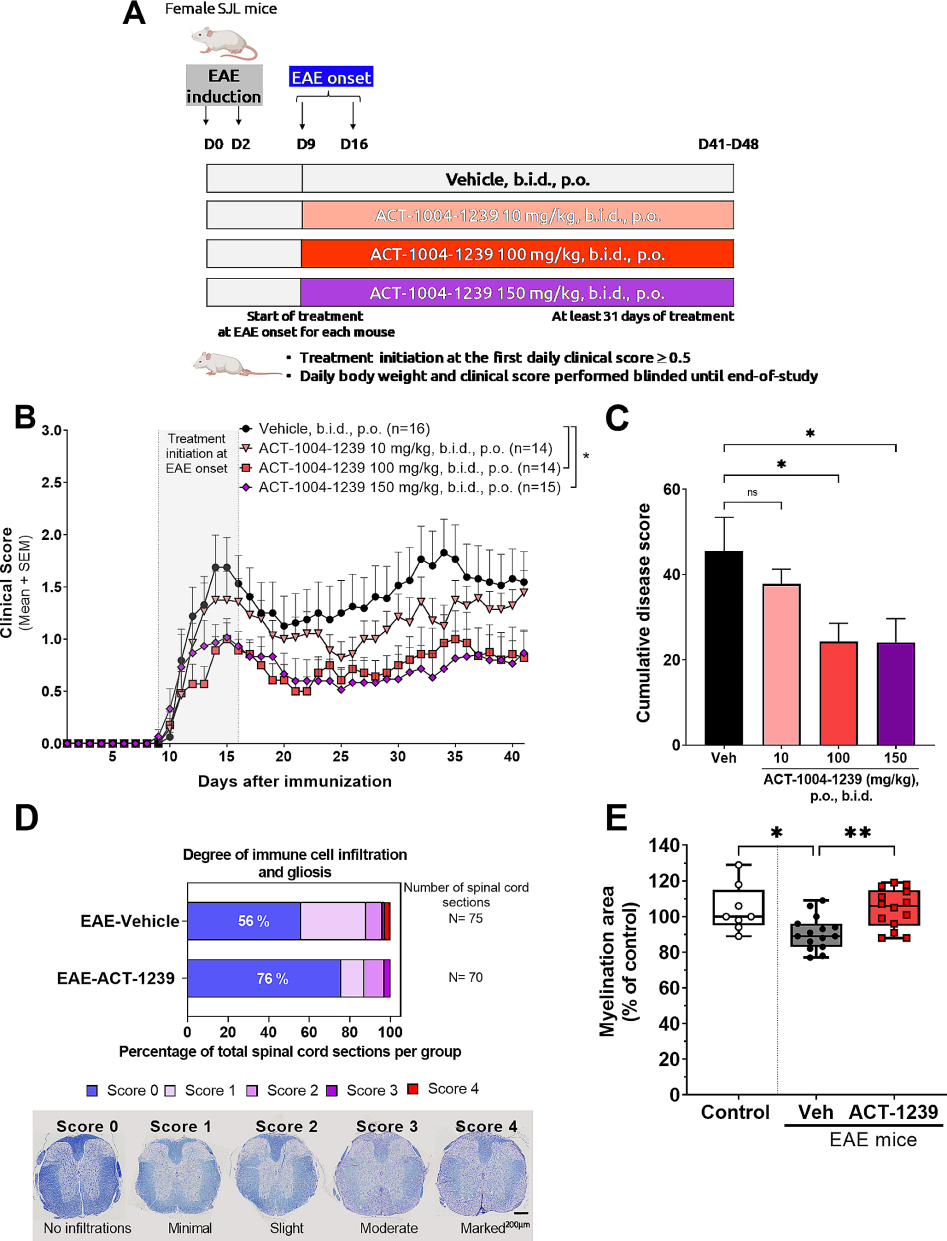
Therapeutic ACT-1004-1239 treatment dose-dependently reduces disease severity in the PLP-induced EAE model. EAE was induced by immunization of female SJL mice with PLP_139 − 151_/CFA and pertussis toxin. Vehicle or ACT-1004-1239 (10, 100, or 150 mg/kg) was given orally (p.o.), twice daily (b.i.d.), starting at disease onset for each mouse (therapeutic mode). (**A**) Study design, created with Biorender.com. Mice were assigned to a treatment group at EAE onset. The main readout was the daily clinical score assessing paralysis, performed in a blinded manner. (**B**) Mean clinical score of EAE mice for each treatment group after PLP_139 − 151_/CFA immunization. Results are expressed as mean + SEM (*n* = 14–16 per group). A two-way ANOVA was performed to analyze the effect of time and treatments on clinical scores. There was a significant interaction between the effects of time and treatment (F_120,2200_=1.4, *p* = 0.003). Simple main effects analysis showed that both independent variables, the time and treatment, had a statistically significant effect on clinical score (*p* < 0.0001 and *p* = 0.024, respectively). Multiple comparisons uncorrected Fisher’s test was then performed, *p < 0.05 compared to vehicle control (**C**) Cumulative disease score, defined as the sum of the clinical scores for each mouse over the 41-days study, in each treatment group. Results are expressed as mean + SEM, *n* = 14–16 per group. *p < 0.05 vs. vehicle-treated EAE mice using a Kruskal-Wallis followed by uncorrected Dunn’s multiple comparisons test. (**D**) At the end of the study, spinal cords from vehicle-treated mice and from mice treated with 100 mg/kg ACT-1004-1239, b.i.d., were scored for degree of inflammation and gliosis as indicated for ≥ 70 sections for each treatment group. Data are shown as percentage of total sections evaluated. Representative images of spinal cord sections stained with Cresyl Violet and Luxol Fast Blue are displayed, as well as criteria used to define histopathological scores. Scale: 200 μm. (**E**) Quantification of the LFB stain (% myelination area) in the spinal cord sections from vehicle-treated mice and from mice treated with 100 mg/kg ACT-1004-1239, b.i.d. Values were normalized to the median value of the non-immunized mice (control) which was set to 100%. *p < 0.05, **p < 0.01 vs. vehicle-treated EAE mice using a Kruskal-Wallis followed by uncorrected Dunn’s multiple comparisons test

Under anesthesia with isoflurane, female SJL/J mice were immunized in the rear flanks by subcutaneous injection (s.c.) of 100 µg PLP_139 − 151_ peptide (Peptides International, Zurich, Switzerland) emulsified 1:1 in Complete Freund’s Adjuvant (CFA) containing 4 mg/mL Mycobacterium tuberculosis (Chondrex, Redmond, WA, USA). On days 0 and 2 postimmunization, mice were injected intraperitoneally (i.p.) with 100 ng of pertussis toxin (List Biological Laboratories, Campbell, CA, USA). Control mice used as benchmark for histology were injected s.c. with phosphate buffer saline (PBS) emulsified 1:1 in incomplete Freund’s Adjuvant (IFA) (Sigma-Aldrich) on day 0. On days 0 and 2, control mice were injected i.p. with PBS. Three independent experiments were conducted, and the study designs are illustrated in Figs. 1A, 4A and 6A. Treatments were initiated either from day 0 after immunization (preventive setting) or from EAE disease onset for each mouse (therapeutic setting).

### Clinical Score Assessment

Mice were weighed, and clinical scores for EAE disease assessment were recorded daily in a blinded manner. Clinical scoring was performed as previously described [6] on a 0-to-5 scale, with 0 = no symptoms, 0.5 = end tail paralysis, 1 = full tail paralysis, 1.5 = one hind limb weakness, 2 = bilateral partial hind limb paralysis, 2.5 = unilateral complete hind limb paralysis, 2.75 = score of 2.5 + unilateral partial hind limb paralysis, 3 = complete bilateral hind limb paralysis, 3.25 = 3 + unilateral partial forelimb paralysis, 3.5 = 3 + unilateral complete forelimb paralysis, 4 = complete paralysis (moribund) and, 5 = death or euthanized. In accordance with the approved animal protocol, mice were euthanized if they reached a score of 4, or if they had a score of 3.5 during three consecutive days, or a score of 3.25 during five consecutive days, or a score of 3 during seven consecutive days.

Relapses were defined as the number of times the clinical score increased by at least 1 point after a period of improvement and after the first peak of the disease. Cumulative disease score was calculated as the sum of the clinical scores for each mouse over the study period.

### Flow Cytometry of the Blood

At the end of the study, after 31 to 33 days of treatment for each mouse, blood from EAE mice was collected and analyzed by flow cytometry. Blood cells were stained with the following surface fluorochrome-conjugated monoclonal anti-mouse antibodies: APC anti-mouse CD25 (BD Biosciences, Franklin Lakes, NJ, USA, Clone PC61), APC-Cy7 anti-mouse CD44 (BD Biosciences, Clone IM7), AF700 anti-mouse CD19 (Biolegend, San Diego, CA, USA, Clone 6D5), PB anti-mouse CD4 (BD Biosciences, Clone RM4-5), PE anti-mouse CD62L (BD Biosciences, Clone Mel-14), PECy7 anti-mouse βTCR (Biolegend, clone H57-597), PerCPCy5.5 anti-mouse CD8 (Biolegend, Clone 53 − 6.7). Staining was performed on ice, in the dark, during 30 min after preincubation with Fc receptor blocker (CD16/CD32, BD Biosciences). Red blood cells were then lysed using RBC lysis buffer (Biolegend) for 10 min, in the dark at room temperature. Dead cells were excluded based on their positive staining with propidium iodide (Sigma-Aldrich). Samples were acquired on Cytoflex flow cytometer (Beckman Coulter Life Sciences, Nyon, Switzerland), and data were analyzed using Kaluza analysis software version 2.1 (Beckman coulter). Cells were first gated in forward scatter versus side scatter, and doublets were excluded based on forward scatter height-area. From the singlet cells, dead cells were excluded based on their positive staining with PI. Cell subsets were quantified among viable cells (singlet cells, PI negative cells): B cells (CD19^+^ cells), T cells (βTCR^+^ cells), CD4^+^ cells (βTCR^+^, CD4^+^ cells), CD8^+^ cells (βTCR^+^, CD8^+^ cells), Naïve T cells (βTCR^+^, CD62L^+^, CD44^−^ cells), Central memory T cells (βTCR^+^, CD62L^+^, CD44^+^ cells), Effector/Effector memory T cells (βTCR^+^, CD62L^−^, CD44^+^ cells).

### Quantification of Plasma CXCL11 and CXCL12 Concentrations

Blood collected in EDTA-coated tubes (BD Microtainer) was centrifuged to prepare plasma samples. After blood collection, mice were transcardially perfused with PBS/EDTA and brains were collected. Brains were homogenized (FastPrep, MP Biomedicals, Illkirch, France) in RIPA lysis buffer supplemented with 1% protease inhibitor (Sigma-Aldrich) and phosphate inhibitor (PhosSTOP Tablets, Roche). Concentrations of CXCL12 and CXCL11 were measured in plasma as previously described [18]. Briefly, plasma and brain CXCL12 concentrations were measured using a commercial Quantikine ELISA mouse CXCL12/SDF1α kit (R&D Systems, Minneapolis, MN, United States). CXCL11 concentrations were quantified using an ultrasensitive immunoassay built on the Single Molecule Counting (SMC™) technology (Erenna® Immunoassay System, Merck Millipore, Billerica, MA, United States).

### Flow Cytometry of the CNS Tissue

To evaluate immune-cell infiltration in the CNS, brain and spinal cord were harvested at day 9 post-immunization, before EAE onset and prepared as neural single cell suspensions and analyzed by flow cytometry. Briefly, mice were euthanized with an overdose of pentobarbital (Esconarkon, Streuli Pharma SA, Uznach, Switzerland), perfused with saline, and spinal cord and right brain hemisphere were isolated and weighed. CNS tissue was homogenized, and CNS cell suspensions were prepared as previously described [6]. CNS cells were stained with the following surface monoclonal anti-mouse antibodies: B220 (BD Biosciences, Clone RA3-6B2), Ly6G (Biolegend, Clone 1A8), CD11b (Biolegend, Clone M1/70), CD3 (Biolegend, Clone 17A2), CD45 (Biolegend, Clone 30-F11), CD11c (Biolegend, clone N418), Ly6C (Biolegend, Clone HK1.4), CD49b (Biolegend, Clone DX5). Staining was performed on ice, in the dark, during 35 min after preincubation with a Fc receptor blocker (CD16/CD32, BD Biosciences). Dead cells were excluded based on their positive staining with propidium iodide (PI, CAS 25535-16-4, Sigma-Aldrich). Cell subsets were quantified among singlets/viable/CD45^+^ cells (PI^−^, CD45^+^ cells) as previously described [6]: neutrophils (CD45^high^, CD11b^+^, Ly6C^+^, Ly6G^high^ cells), monocytes (CD45^high^, CD11b^+^, Ly6G^−^, Ly6C^high^ cells), microglia (CD11b^+^, Ly6G^−^, Ly6C^−^, CD45^low^ cells), monocyte-derived cells (MdCs) (CD45^high^, CD11b^+^, Ly6G^−^, Ly6C^−^cells), B cells (CD45^high^ CD11b^−^, Ly6G^−^, Ly6C^−^, B220^+^ cells), plasmacytoid dendritic cells (pDCs) (CD45^high^, CD11b^−^, Ly6G^−^, Ly6C^+^, B220^+^, CD11c^+^ cells), T cells (CD45^high^, CD11b^−^, B220^−^, CD49b^−^, CD3^+^ cells), natural killer (NK) T cells (CD45^high^, CD11b^−^, B220^−^, CD49b^+^, CD3^+^ cells), NK cells (CD45^high^ CD11b^−^, B220^−^, CD49b^+^, CD3^−^ cells), and dendritic cells (DCs) (CD11b^−/low^, B220^−^, CD49b^−^, CD3^−^, CD11c^+^ cells). Results were expressed as the percentage of total CD45^+^ cells, including the microglia.

### Flow Cytometry of the Lymph Nodes

To evaluate T cell activation and polarization, cervical lymph nodes were harvested at day 9 post-immunization, prepared as single cell suspensions, and counted using Vi-Cell viability analyzer (Beckman coulter). Cells (2*10^6^ cells/well) were stimulated with PMA (Sigma-Aldrich) and ionomycin (Sigma-Aldrich) at 10 ng/mL and 1 µg/mL final, respectively in the presence of DNase I (Sigma-Aldrich) at 20 µg/mL final, GolgiPlug and GolgiStop (BD Biosciences) at 37 °C in an incubator, overnight. After the stimulation, cells were incubated on ice with a Fc receptor blocker (CD16/CD32, BD Biosciences) and then stained with the following surface fluorochrome conjugated monoclonal anti-mouse antibodies: CD11b (Biolegend, Clone M1/70), CD19 (Biolegend, Clone 6D5), together with Acqua Dye live/dead dye (Invitrogen-Thermo Fisher Scientific). Staining was performed on ice, in the dark, for 20 min. Cells were then fixed and permeabilized using Cytofix/Cytoperm solution (BD Biosciences). The intracellular cytokine stain was carried out using the following antibodies diluted in permeabilization buffer (BD Biosciences): IL-17 A (Biolegend, Clone TC11-18H10.1), CD3 (BioLegend, Clone 17A2), IFN-γ (Biolegend, Clone XMG1.2), TNF-α (Biolegend, Clone MP6-XT22), IL-10 (BD Biosciences, Clone JES5-16E3), CD69 (BD Biosciences, Clone H1.2F3). Flow cytometry was performed on a CytoFLEX flow cytometer (Beckman Coulter Life Sciences, Nyon, Switzerland) and analyzed using Kaluza analysis software version 2.1 (Beckman Coulter). Results were expressed as percentages of CD69^+^ T cells in lymph nodes.

### Transwell Cell Migration in Vitro Assay

Spleens from PLP-induced EAE mice were collected 23 or 32 days after immunization. Spleens were smashed and processed as single cell suspensions. Red blood cells were then lysed using RBC lysis buffer (Biolegend) for 5 min in the dark at room temperature. The study was divided into four independent experiments using four different preparations of cells (*n* = 4 mice). Each condition/mouse was performed in triplicates. Splenocytes were resuspended in serum-free medium (Panserin P401, Pan-Biotech, Aidenbach, Germany) and stained with 3µM of Calcein-AM (Invitrogen-Thermo Fisher Scientific) for 30 min at 37 °C, and 5% CO_2_. After washing, stained cells were incubated with or without test compounds for 30 min at 37 °C. The ACKR3 antagonist ACT-1004-1239, the positive control CXCR4 antagonist, AMD3100 (sigma-Aldrich), and the CXCR3 antagonist AMG287 [19] (Chembiotek Research International, Telangana, India) were applied at concentrations of 500 nM and 1 µM for 30 min at 37 °C, and 5% CO_2_. Mouse chemoattractant (30µL/well), either murine CXCL11 or murine CXCL12 (Peprotech, Cranbury, NJ, USA), diluted in the medium at the final concentrations given in the figure legends, was added to the bottom chamber of the 3µM pore size ChemoTx® disposable plate (Neuro Probe, Gaithersburg, MD, USA). Stained cell suspensions (200 000 cells/25µL/well) preincubated with or without test compounds were pipetted on top of the filter membrane of the transwell insert. Following a 1h30 incubation at 37 °C with 5% CO2, the quantification of migrated cells in the lower chamber of the microplate was conducted through fluorescence analysis using the ImageXpress Micro XL cell imaging system (Molecular Devices, San Jose, CA, USA). In each experiment, a non-treated control condition with chemoattractant (Medium) and a non-treated control condition with no chemoattractant (no CXCL12 or no CXCL11) were added to the plate.

### Cuprizone-Induced Demyelination Model

Cuprizone (Sigma- Aldrich) was prepared as food admix in the regular food pellet 3336 (Granovit AG) at a final concentration of 0.2% of food. Due to cuprizone instability, fresh new food was placed in the feeder twice per week. Male C57BL/6 mice were exposed to cuprizone via food admix for 5 or 6 weeks (demyelination phase). Cuprizone food was then removed, and mice were allowed to recover for another 1 or 2 weeks (remyelination phase). Study designs are illustrated in Fig. 7A and F. Mice were randomized based on their body weight in the different treatment groups. Clemastine, an antihistaminic drug with antimuscarinic properties approved by the FDA, is known for its ability to promote remyelination in preclinical demyelination models such as the cuprizone model [19]. Having successfully completed a small Phase 2 study in patients with MS [20], clemastine was employed as a positive control in this model. Treatments with vehicle (b.i.d.), ACT-1004-1239 (100 mg/kg, b.i.d.) or clemastine fumarate (10 mg/kg, q.d.) were initiated after 5 weeks of cuprizone exposure.

### Histological Analysis

At the end of study, mice were euthanized with pentobarbital (Esconarkon, Streuli Pharma SA) and transcardially perfused with a solution of PBS/EDTA 0.5 mM (Invitrogen) followed by a second perfusion with 4% paraformaldehyde (PFA, Biosystem) in saline. Brains or spinal cords were isolated as a whole and immersed in a cassette in 4% PFA for 24 h, dehydrated with alcohol, and embedded in paraffin wax. Coronal paraffin sections of 2 μm thickness were cut from the frontal brain or from five regions of the spinal cord in the cuprizone model or EAE model, respectively. Sections were stained with cresyl violet and luxol fast blue (CV-LFB) for evidence of demyelination as previously described [6].

Semiquantitative histological evaluation was performed and scored by a histopathologist in a blinded fashion. From each mouse, 5 sections of the spinal cord were examined, and histopathological scores based on inflammatory cell infiltration, gliosis, and meningitis, were defined as follows: 0, normal; 1, minimal; 2, slight; 3, moderate; and 4, marked (Fig. 1D).

### Immunohistochemistry

Paraffin Coronal brain sections of 4 μm were stained using Leica Bond RX (Leica Microsystems, Milton Keynes, UK) automated stainer with optimized staining protocols. Microglia and astrocytes were identified by IBA1 (Fujifilm Wako Chemicals, Richmond, VA, USA, #019-19741) and GFAP (Dako, Agilent Santa Clara, CA, USA, #Z0334), respectively. Multiplex IHC using Tyramide Signal Amplification (TSA) were applied and 2 fluorophores were used: TSA Plus Cyanine 3.5. and Cyanine 5.5 (Akoya Biosciences, Marlborough, MA, USA). Counterstain DAPI (Sigma, #D-9542).

### Image Acquisition and Quantification

The slides were scanned using S60 NanoZoomer whole-slide scanner (Hamamatsu Photonics, Solothurn, Switzerland). Images were acquired at 20x magnification for chromogenic staining and 40x for fluorescently labelled staining. The images were uploaded onto ORBIT image analysis platform (Version 3.15) for quantification of the positive area out of the total area of interest, expressed as percentage as previously described [6].

### Quantification of Plasma and Brain ACT-1004-1239 Concentrations

Concentrations of ACT-1004-1239 in plasma and brain tissue were measured using liquid chromatography coupled to tandem mass spectrometry (LC-MS/MS). The bioanalytical method used for the quantification of ACT-1004-1239 included the use a deuterated internal standard. Quality control samples with an acceptance criteria of ± 15% in the bioanalytical runs were used to check the performance of the method. The lower limit of quantification (LLOQ) is specified in the figure legends.

### Statistical Analysis

All statistical analysis were performed using Prism version 9 (Graph Pad Prism software, San Diego, CA, USA) using the tests specified in the figure legends. Differences were considered significant at *p* < 0.05.

## Results

### ACKR3 Antagonism with ACT-1004-1239 Reduces PLP-Induced EAE Disease Severity

The therapeutic potential of ACKR3 antagonism on established EAE disease was evaluated in the relapsing-remitting PLP-induced EAE model in female SJL mice. This model is commonly used for evaluating the effectiveness of immunomodulatory agents, but it has also been employed to determine the efficacy of promyelinating agents, which induce OPC differentiation [21, 22]. Treatment with the ACKR3 antagonist ACT-1004-1239 (10, 100 and 150 mg/kg, b.i.d.) or vehicle was initiated for each mouse at the first sign of disease (clinical score ≥ 0.5), between 9 and 16 days after immunization (Fig. 1A). Treatment groups were randomized for similar body weight, clinical score, and disease onset day (Supplementary Fig. 1A).

In line with published data [23], vehicle-treated mice exhibited a typical relapsing-remitting (RR) disease course with a first peak of disease 14 days after immunization (one day after treatment initiation), followed by spontaneous remission and a second peak 34 days after immunization (Fig. 1B and supplementary Fig. 1B). Therapeutic treatment with ACT-1004-1239 at 100 mg/kg or 150 mg/kg, b.i.d., significantly decreased the severity of EAE disease, maintaining a mean clinical score below 1 from day 4 after treatment initiation to the end of the study (Fig. 1B and supplementary Fig. 1B). Both doses reduced significantly and similarly the cumulative disease score, representing the overall extent of the disease, as compared to EAE vehicle-treated mice (*p* = 0.027 and *p* = 0.011, respectively) (Fig. 1C). Furthermore, ACT-1004-1239 at 100 and 150 mg/kg, b.i.d., reduced body weight loss compared to EAE vehicle-treated mice (Supplementary Fig. 1C). Therapeutic treatment with 10 mg/kg ACT-1004-1239, b.i.d., resulted in slightly lower mean clinical scores throughout the study as compared to vehicle-treated EAE mice, without reaching statistical significance on the cumulative disease score (*p* = 0.870) (Fig. 1B-C and supplementary Fig. 1B-C).

To further study the efficacy of ACT-1004-1239 on neuroinflammation and demyelination, spinal cord sections from EAE groups treated with either vehicle or with ACT-1004-1239 at 100 mg/kg, b.i.d., were stained with Cresyl Violet and Luxol Fast Blue to assess neuroinflammation and myelination, respectively. At the end of the study, therapeutic treatment with ACT-1004-1239 improved neuroinflammation as shown by a trend in the reduction of immune cell infiltration and gliosis compared to vehicle-treated EAE mice (Fig. 1D and supplementary Fig. 1D-1E). Furthermore, therapeutic administration of ACT-1004-1239 significantly reduced EAE-induced demyelination as shown by increased myelination area vs. vehicle-treated EAE mice (105.8% myelination in ACT-1004-1239-treated EAE mice vs. 88.7% in vehicle-treated EAE mice; *p* = 0.0027) (Fig. 1E). At this stage of the disease, while there was a significant positive correlation between the histopathological scores determined by a pathologist using CV-LFB staining and the clinical scores assigned to each mouse (supplementary Fig. 1F; Spearman *r* = 0.76, *p* < 0.0001), this correlation was not observed with the quantification of T cells area in the spinal cord from EAE mice (supplementary Fig. 1G).

### Efficacy of ACT-1004-1239 in EAE is Associated with both an Increase in CXCL11 and CXCL12 Plasma Concentrations

**Fig. 2:**
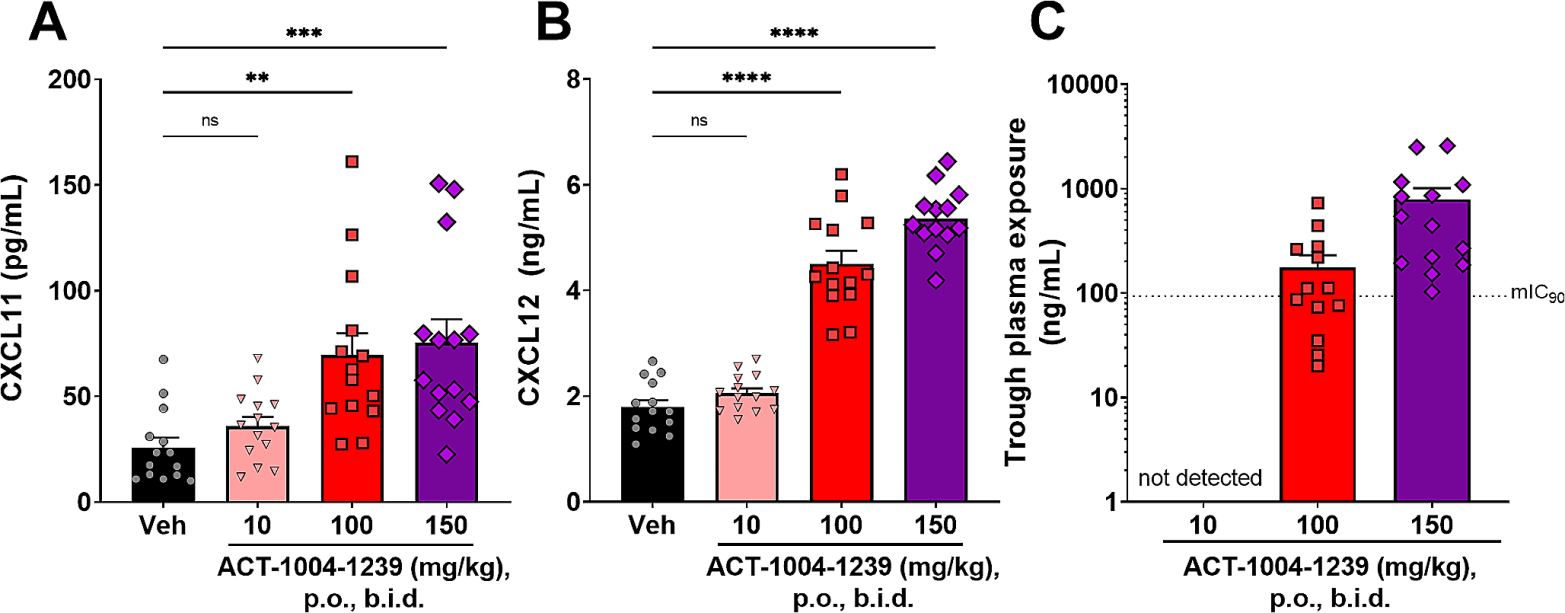
ACT-1004-1239 dose-dependently increases plasma CXCL11 and CXCL12 concentrations in PLP-EAE mice. EAE was induced by immunization of female SJL mice with PLP_139 − 151_/CFA and pertussis toxin. Vehicle or ACT-1004-1239 (10, 100, or 150 mg/kg) was given orally (p.o.), twice daily (b.i.d.), starting at disease onset for each mouse (therapeutic mode). Plasma samples were prepared at sacrifice, after 31–33 days of treatment for each mouse, 14 h after last dosing (at trough). (**A**) CXCL11 and (**B**) CXCL12 plasma concentrations at trough. Results are expressed as mean + SEM (*n* = 13–14 group). One-way ANOVA was performed to compare the effect of treatment on chemokines plasma concentrations. There was a statistically significant difference in both CXCL11 and CXCL12 levels between at least two groups (F_3,52_=9.134, *p* < 0.0001 and F_3,51_=112.4, *p* < 0.0001, respectively). Dunnett’s multiple comparisons test was then performed ** *p* < 0.01, ***p < 0.001, ****p < 0.0001 versus vehicle-treated EAE mice (**C**) ACT-1004-1239 plasma concentrations measured at trough, expressed as mean + SEM (*n* = 13–14 per group). The dotted line represents the concentration inhibiting 90% of ACKR3 molecules (mouse IC_90_) based on an in vitro β-arrestin recruitment assay

CXCL11 and CXCL12 levels, controlled by ACKR3 scavenging activity, has been proposed to be one of the mechanisms by which antagonism of ACKR3 could exert therapeutic effects [18]. Administration of ACT-1004-1239 in the PLP-induced EAE model resulted in a dose-dependent increase in both plasma CXCL11 and CXCL12 concentrations measured at trough, at the end of the study (Fig. 2A-B). The administration of the highest tested doses (100 and 150 mg/kg of ACT-1004-1239, b.i.d.) led to mean drug plasma concentrations at trough, i.e. 14 h after the last dosing occasion, capable of inhibiting over 90% of mouse ACKR3 molecules (mIC_90_) based on the in vitro β-arrestin recruitment assay described earlier [17] (Fig. 2C). Interestingly, no further increase in efficacy was achieved at doses ≥ 100 mg/kg, twice daily, in this model (Fig. 1B).

### Treatment with ACT-1004-1239 Dose-Dependently Increases Blood Lymphocytes

**Fig. 3:**
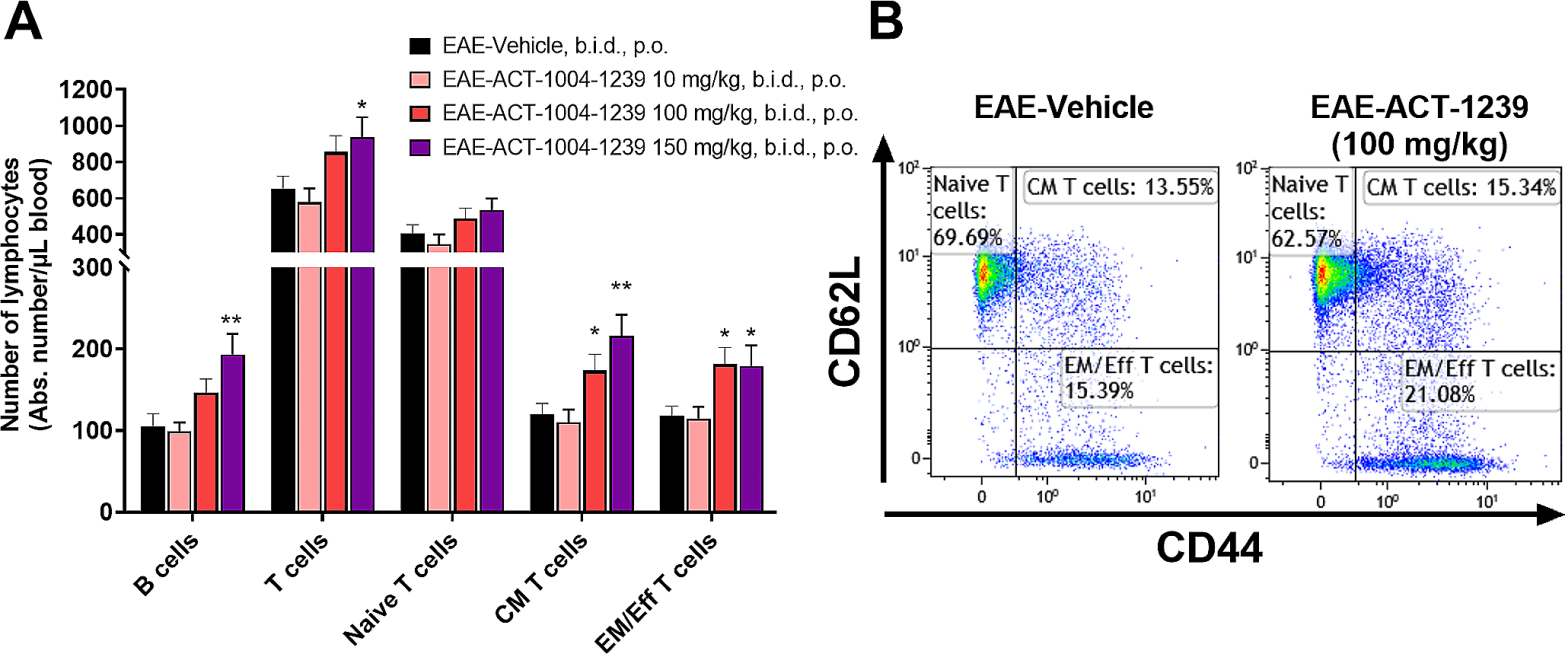
ACT-1004-1239 dose-dependently increases blood lymphocyte counts in the PLP-induced EAE model EAE was induced by immunization of female SJL mice with PLP_139 − 151_/CFA and pertussis toxin. Vehicle or ACT-1004-1239 (10, 100, or 150 mg/kg) was given orally (p.o.), twice daily (b.i.d.), starting at disease onset for each mouse (therapeutic mode). Blood samples were collected at sacrifice, after 31 to 33 days of treatment for each mouse, 14 h after the last oral gavage, at trough. Blood cell subpopulations were analyzed by flow cytometry. (**A**) Lymphocyte subtype counts are expressed as mean + SEM (*n* = 14–16 per group). One-way ANOVA was performed to compare the effect of treatment on lymphocyte counts. There was a statistically significant difference in both overall T and B cell counts between at least two treatment groups (F_3,52_=3.683, *p* = 0.018 and F_3,52_=6.139, *p* = 0.001, respectively). *p < 0.05, **p < 0.01, using Dunnett’s multiple comparisons test. (**B**) Representative flow cytometry plots from vehicle-treated and ACT-1004-1239 100 mg/kg, b.i.d.-treated EAE mice depicting the gating strategy among T cells (CD3^+^) for naïve T cells (CD62L^+^CD44^−^), central memory (CM) T cells (CD62L^+^CD44^+^), and effector memory/effector (EM/Eff) T cells (CD62L^−^CD44^+^)

The primary pathological immunological processes involved in MS and EAE are thought to be lymphocytes mediated, therefore most of MS treatments focus on lymphocytes [24]. Unlike the majority of currently approved immunomodulatory treatments for MS, which typically induce lymphopenia [24], the administration of ACT-1004-1239 resulted in an elevation of blood B and T lymphocyte counts in EAE mice (Fig. 3A). Therapeutic administration of ≥ 100 mg/kg ACT-1004-1239, b.i.d., dose-dependently and significantly increased central memory and effector memory/effector T cells vs. vehicle-treated EAE mice (Fig. 3A-B). Interestingly, absolute T and B lymphocyte counts moderately correlated with plasma CXCL12 concentrations (Supplementary Fig. 2A-B).

### ACT-1004-1239 Reduces CNS Leukocyte Infiltrates in EAE Mice without Affecting Peripheral T Cell Activation nor Polarization

**Fig. 4:**
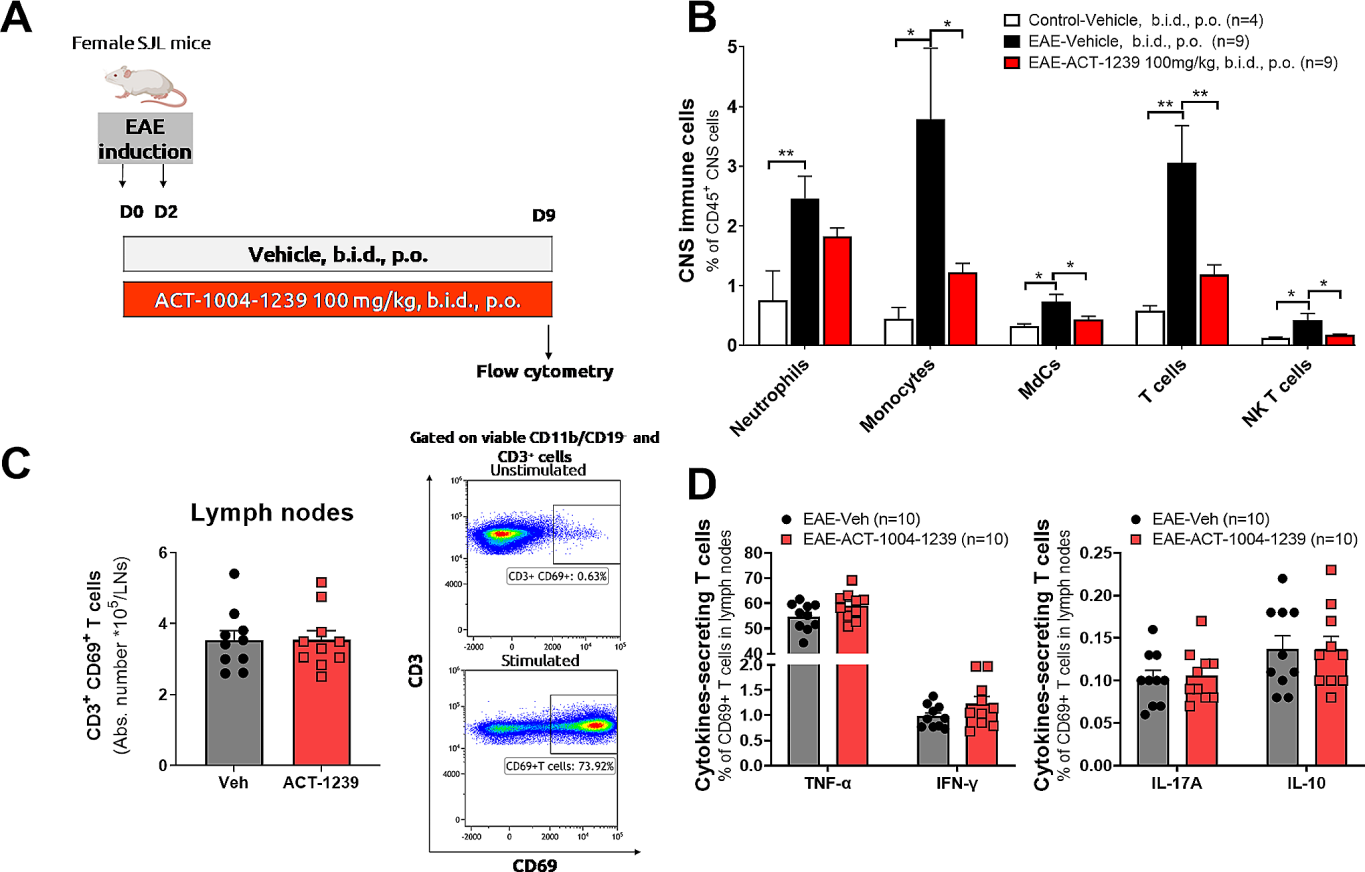
ACT-1004-1239 reduces CNS immune cells infiltration without affecting T cell activation in draining lymph nodes EAE was induced by immunization of female SJL mice with PLP_139 − 151_/CFA and pertussis toxin. Vehicle or ACT-1004-1239 (100 mg/kg) was given orally (p.o.), twice daily (b.i.d.), starting at PLP_139 − 151_/CFA immunization (*n* = 10/group). Negative control mice were injected with PBS/IFA. Mice were sacrificed before appearance of clinical signs, 9 days after immunization. (**A**) Study design (**B**) Right brain hemisphere and spinal cord of each mouse were collected and processed as neural single cell suspensions for flow cytometry analysis. ACT-1004-1239 reduces immune cell infiltrates into the CNS. MdCs: monocyte-derived cells. Results are expressed as mean + SEM. * *p* < 0.05, **p < 0.01 using one-way ANOVA followed by uncorrected Fisher’s test. (**C**) Cervical lymph nodes were collected and processed as single cell suspensions, counted, and plated at 2*10^6^ cells/condition with or without PMA/ionomycine stimulation overnight and analyzed by flow cytometry. Absolute counts of CD69^+^ T cells in cervical lymph nodes from vehicle- or ACT-1004-1239 treated mice after stimulation. Representative plots of unstimulated and stimulated conditions. (**D**) ACT-1004-1239 does not affect the proportion of TNF-α, IFN-γ, IL-17 A and IL-10-secreting T cells in the draining lymph nodes. Results are expressed as mean + SEM

Next, the impact of ACKR3 antagonism on CNS infiltration of different leukocyte populations was evaluated in the PLP-EAE mouse model (Fig. 4A). Preventive treatment with ACT-1004-1239 significantly reduced CNS infiltrating monocytes, monocyte-derived cells (MdCs), T cells, and NK T cells vs. vehicle-treated EAE mice, confirming the immunomodulatory effect of ACKR3 antagonism in this model (Fig. 4B). To examine a potential effect of ACKR3 antagonism on T cell activation and polarization, numbers of activated T cells in draining lymph nodes and their cytokine-secretion profile were assessed. (Fig. 4C-D). ACT-1004-1239 did not affect ex-vivo T-cell activation as shown by comparable numbers of CD3^+^CD69^+^ T cells in ACT-1004-1239- and vehicle-treated animals (Fig. 4C). In addition, ACKR3 antagonism did not impact cytokine-secretion of ex-vivo stimulated T cells; comparable proportions of cytokine-secreting T cells (TNF-a, IFN-g, IL-17 A, IL-10) were measured in mice treated with the ACKR3 antagonist or vehicle (Fig. 4D).

### ACT-1004-1239 Does Not Affect CXCL12-Induced Chemotaxis in Vitro

**Fig. 5:**
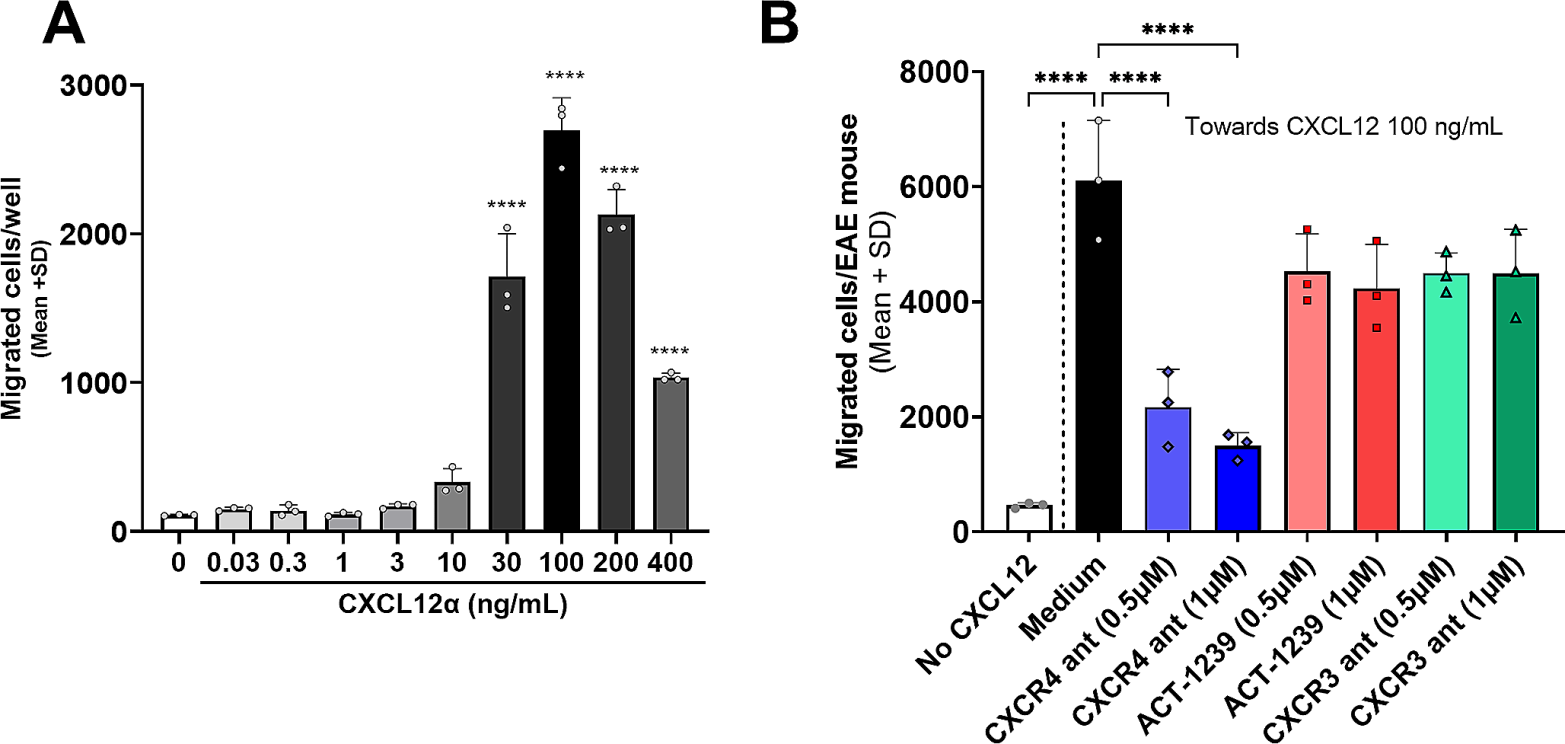
ACT-1004-1239 does not affect directly CXCL12-induced chemotaxis in vitro. EAE was induced by immunization of female SJL mice with PLP_139 − 151_/CFA and pertussis toxin. (**A**) Spleens were collected, processed, and assessed in an in vitro CXCL12 migration assay. Results are expressed as mean + SD, (triplicates/condition). (**B**) CXCR4 antagonism (CXCR4 ant) with AMD3100 reduces dose-dependently CXCL12 (100 ng/mL)-induced chemotaxis of splenocytes extracted from EAE mice. ACKR3 or CXCR3 antagonism (ACT-1004-1239 or AMG287, respectively) did not affect splenocyte migration. Results are expressed as mean + SD. (Each condition was performed in triplicates, which were averaged for each mouse; *n* = 3 mice). ****p < 0.0001 using one way ANOVA followed by Dunnett’s multiple comparisons test

The direct role of ACKR3 in leukocyte migration is widely debated, as it seems to differ depending on the microenvironment and cellular context [11, 12, 25]. To determine the extent to which the efficacy of ACT-1004-1239 in the PLP-induced EAE model results from a direct effect on CXCL12-dependent leukocyte migration, an in vitro migration assay was conducted. First, the migration of freshly isolated splenocytes from EAE mice in response to different concentrations of CXCL11 and CXCL12 was assessed. Splenocytes demonstrated significant migration towards CXCL12 at concentrations ≥ 30 ng/mL, displaying a typical bell-shaped dose response curve (Fig. 5A). No significant migration was observed towards CXCL11 at the dose range tested without prior ex vivo activation of the cells (Supplementary Fig. 3A-B). Maximum splenocyte migration from EAE mice was observed in response to CXCL12 at a concentration of 100 ng/mL (Fig. 5A). This in vitro CXCL12-induced chemotaxis was dose-dependently reduced by AMD3100, a CXCR4 antagonist, which was included as a positive assay control. However, ACT-1004-1239 and AMG487, a CXCR3 antagonist [19] used as a negative control in this assay, did not significantly reduce splenocyte migration at the tested doses (Fig. 5B). These results suggest that in vitro leukocyte chemotaxis towards CXCL12 is regulated trough the CXCL12/CXCR4 axis and not directly through the CXCL12/ACKR3 axis.

### ACT-1004-1239 Combined with Siponimod Further Reduces EAE Disease Severity

**Fig. 6:**
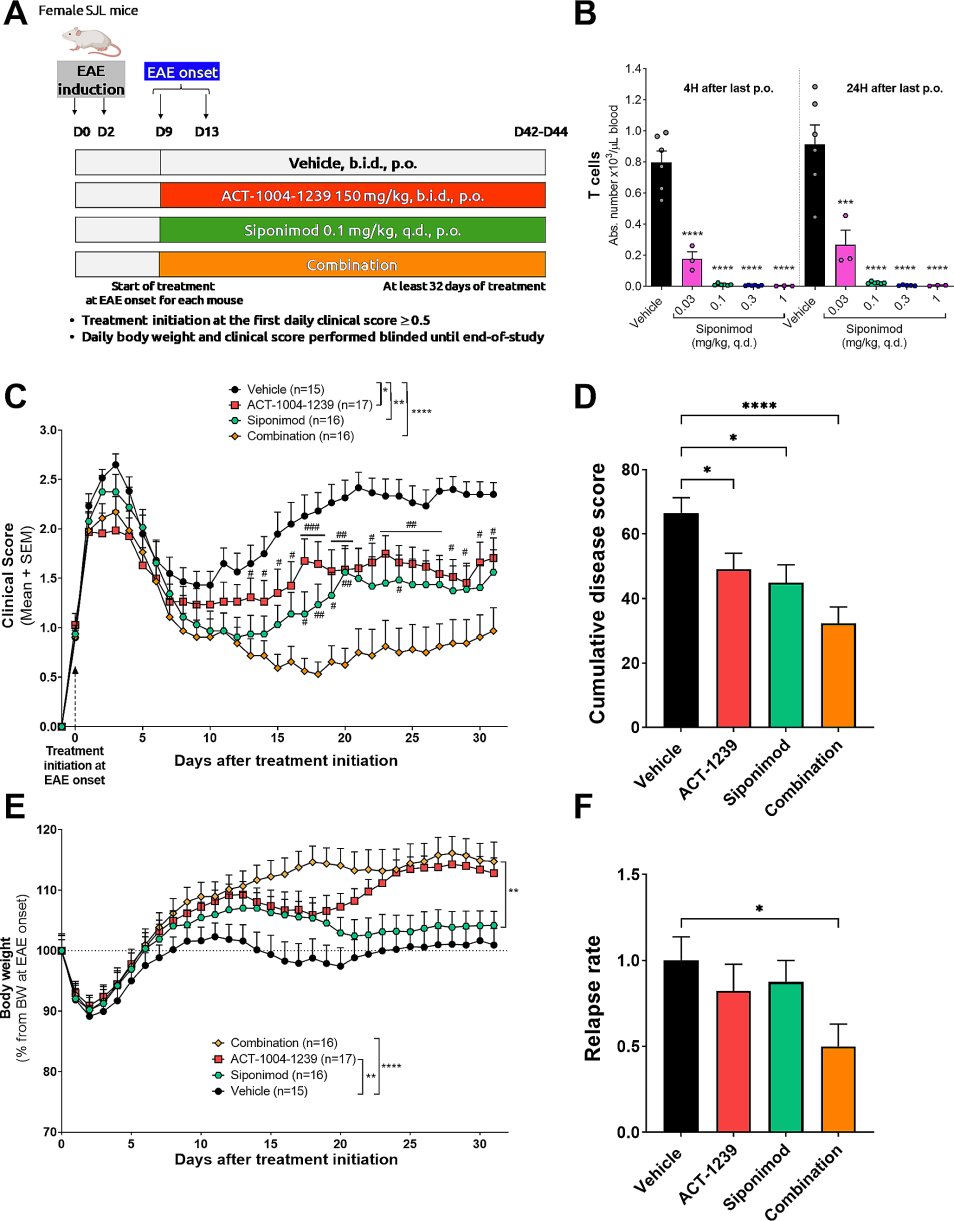
Combination of ACT-1004-1239 with siponimod further reduces EAE disease severity compared to each monotherapy. EAE was induced by immunization of female SJL mice with PLP_139 − 151_/CFA and pertussis toxin. Vehicle, ACT-1004-1239 (150 mg/kg, twice daily (b.i.d.)), siponimod (0.1 mg/kg, once daily (q.d.)), or the combination of ACT-1004-1239 with siponimod was given orally (p.o.), starting at disease onset for each mouse (therapeutic mode). (**A**) Study design, created with Biorender.com (**B**) Naive SJL mice were treated for 3 days with vehicle or Siponimod (0.03, 0.1, 0.3, or 1 mg/kg). Blood was collected 4–24 h after the last oral dose and processed for flow cytometry. Absolute counts of T cells are expressed as mean + SEM (*n* = 3–6/group). ****p < 0.0001 using one way ANOVA followed by Dunnett’s multiple comparisons test versus vehicle-treated mice. (**C**) Mean clinical score of EAE mice for each treatment group after treatment initiation. Results are expressed as mean + SEM (*n* = 15–17 per group). A two-way ANOVA was performed to analyze the effect of time and treatments on clinical scores. There was a significant interaction between the effects of time and treatment (F_96,1920_=3.953, *p* < 0.0001). Simple main effects analysis showed that both independent variables, the time and treatment, had a statistically significant effect on clinical score (*p* < 0.0001 and *p* = 0.0001, respectively). Multiple comparisons uncorrected Fisher’s test was then performed, *p < 0.05, **p < 0.01, ***p < 0.001 compared to vehicle control. #*p* < 0.05, ##*p* < 0.01, ###*p* < 0.001 using t-test versus combination-treated group. (**D**) Cumulative disease index, defined as the sum of the clinical scores for each mouse over the 42-days study, in each treatment group. Results are expressed as mean + SEM, *n* = 15–17 per group. *p < 0.05, ****p < 0.0001 vs. vehicle-treated EAE mice using a Kruskal-Wallis followed by uncorrected Dunn’s multiple comparisons test. (**E**) Body weight evolution after treatment initiation. Results are expressed as the mean percentage of the body weight of each mouse at the time of treatment initiation, which was set to 100%. A mixed-effects analysis was performed to analyze the effect of time and treatments on body weight. There was a significant interaction between the effects of time and treatment (F_93,1849_=5.864, *p* < 0.0001). Simple main effects analysis showed that both independent variables, the time and treatment, had a statistically significant effect on body weight (*p* < 0.0001 and *p* < 0.0001, respectively). **p < 0.01, ****p < 0.0001 using Tukey’s multiple comparisons test. (**F**) Relapse rate. Results are expressed as mean + SEM (*n* = 15–17/group). *p < 0.05 vs. vehicle-treated EAE mice using a Kruskal-Wallis followed by uncorrected Dunn’s multiple comparisons test

In view of the complex pathogenesis and progressive nature of MS, combination therapy may improve disease control over monotherapy. Siponimod, approved for the treatment of patients with RRMS and secondary progressive MS, is a selective sphingosine 1-phosphate (S1P) 1,5 receptor modulator. Siponimod inhibits lymphocyte egress from lymphoid tissue into the blood, leading to lymphopenia, and prevents the infiltration of auto-reactive lymphocytes into the CNS [24]. To assess the efficacy of ACT-1004-1239 in comparison to siponimod, and to investigate the potential synergistic clinical benefits of combining ACT-1004-1239 with siponimod, both monotherapies and their combination were evaluated in the PLP-EAE model. Starting at onset of EAE for each mouse, between 9 and 13 days after immunization, mice were randomized and treated orally with vehicle, ACT-1004-1239 monotherapy (150 mg/kg, b.i.d.), siponimod monotherapy (0.1 mg/kg, q.d.), or the combination of ACT-1004-1239 with siponimod (Fig. 6A). In preclinical EAE models, siponimod has shown to be highly efficacious at doses inducing maximal lymphopenia [26, 27]. Oral siponimod at 0.1 mg/kg led to maximal reduction of blood lymphocyte counts over 24 h in SJL mice (Fig. 6B). In the PLP-EAE model, therapeutic treatment with ACT-1004-1239 or siponimod similarly and significantly reduced signs of paralysis in mice (Fig. 6C). Mice administered with the combination therapy exhibited significantly lower mean clinical scores than vehicle- or monotherapy-treated mice. Only the combination treatment maintained a mean clinical score below 1 from day 9 after treatment initiation. In addition, both monotherapy treatments exhibited a comparable reduction in the overall extent of the disease, and this decrease was further enhanced by the combination (*p* < 0.0001 versus vehicle-treated EAE mice) (Fig. 6D). While siponimod had no significant impact on EAE-induced body weight loss, administration of ACT-1004-1239 alone and in combination with siponimod significantly reduced body weight loss over the study duration compared to vehicle-treated EAE mice (*p* = 0.002 and *p* < 0.0001, respectively) (Fig. 6E). Combination of ACT-1004-1239 with siponimod also conferred a synergistic effect on the relapse rate, as shown by a significant relapse rate reduction compared to vehicle-treated mice (*p* = 0.014) (Fig. 6F). As previously shown in naïve SJL mice, maximal lymphocyte count reduction was also seen with siponimod monotherapy and the combination treatment in PLP-induced EAE mice at trough (Supplementary Fig. 4A-B). Furthermore, the plasma siponimod concentrations at trough, 24 h after the last administration, were not significantly affected by the combination therapy (Supplementary Fig. 4C). Importantly, the combination treatment did not affect ACT-1004-1239-induced CXCL11 and CXCL12 plasma increase nor trough plasma exposure (Supplementary Fig. 4D-E-F). Unlike siponimod, the use of ACT-1004-1239 as a monotherapy treatment did not result in lymphopenia (Supplementary Fig. 4G-H).

### ACT-1004-1239 Enhances Remyelination in the Cuprizone-Induced Demyelination Model

**Fig. 7:**
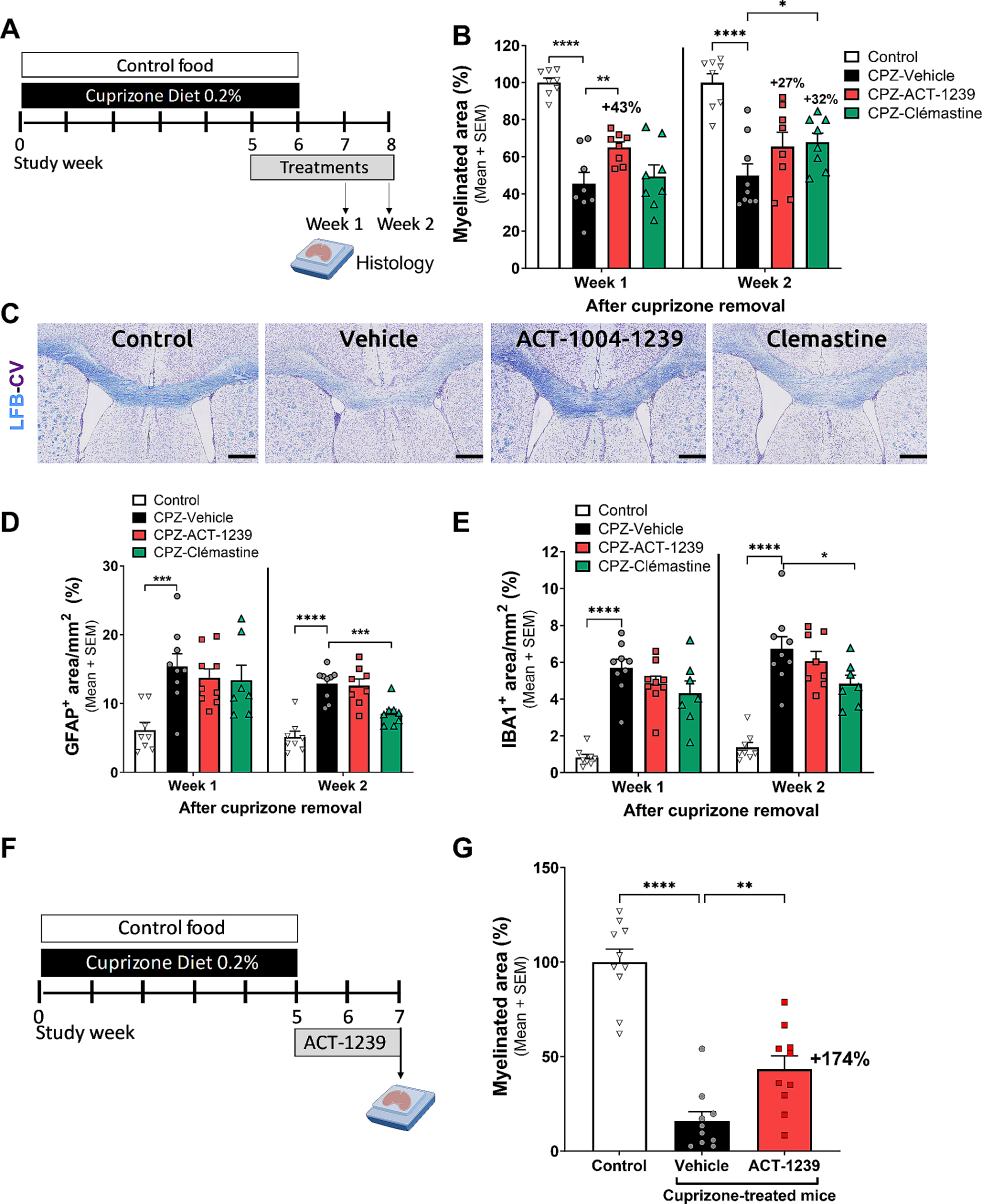
ACT-1004-1239 enhances remyelination in the cuprizone model. Male C57BL/6 mice were exposed to control food (control) or cuprizone diet 0.2% (CPZ) for 6 weeks and then to normal chow for subsequent 2 weeks. Vehicle, ACT-1004-1239 (100 mg/kg, twice daily), or clemastine (10 mg/kg, once daily) were given orally, after 5 weeks of CPZ exposure for 2 or 3 weeks. (**A**) Study design, created with BioRender.com. Representatives of each group (*n* = 8–9/group) were euthanized one or two weeks after CPZ removal. (**B**) Quantitative analysis of myelinated areas in the corpus callosum, 1 or 2 weeks after cuprizone removal. Results are expressed as percentage of the mean positive myelinated area normalized to the mean myelinated area in control, set to 100% for each independent time point; *n* = 8–9 mice in each group. For each time point, a one-way ANOVA was performed to compare the effect of the different treatments on the myelinated area. There was a significant difference in myelinated area between at least two treatment groups (F_3,28_=27.08, *p* < 0.0001, at week 1 and F_3,29_=12.03, *p* < 0.0001 at week 2). Multiple comparisons uncorrected Fisher’s test was then performed, *p < 0.05, **p < 0.01, ****p < 0.0001 compared to vehicle control. (**C**) Representative Luxol Fast Blue-Cresyl Violet (LFB-CV) images of the corpus callosum sections from each treatment group, 1 week after CPZ removal; scale bar 380 μm. ACT-1004-1239 did not impact cuprizone-induced astrogliosis (**D**) and microgliosis (**E**) in the corpus callosum. Quantitative analysis of GFAP (**D**) and IBA1 (**E**) are expressed as the percentage of the mean positive area, normalized by the selected region of interest in mm^2^; *n* = 7–9 mice in each group. A One-way ANOVA was performed to compare the effect of the different treatments on astrogliosis and microgliosis. There was a significant difference in GFAP^+^ area and IBA1^+^ area between at least two treatment groups (F_3,29_=6.33, *p* = 0.002 and F_3,29_=22.50, *p* < 0.0001 at week 1, respectively; F_3,29_=20.76, *p* < 0.0001 and F_3,28_=22.2, *p* < 0.0001 at week 2). Multiple comparisons uncorrected Fisher’s test was then performed *p < 0.05, ***p < 0.001, ****p < 0.0001 versus vehicle-treated CPZ mice. In a second independent experiment, mice were treated orally with vehicle or ACT-1004-1239 (100 mg/kg), twice daily for two weeks, after 5 weeks of CPZ exposure. (**F**) Study design (**G**) Quantitative analysis of myelinated areas in the corpus callosum, 2 weeks after cuprizone removal. Results are expressed as percentage of the mean positive LFB^+^ area normalized to the mean % in mice fed with control food, set to 100% for each study week; *n* = 10 mice/group. A One-way ANOVA was performed to compare the effect of the different treatments on the myelinated area. There was a significant difference in myelinated area between at least two treatment groups (F_3,27_=46.19, *p* < 0.0001). **p < 0.01, ****p < 0.0001 using Dunnett’s multiple comparisons test versus CPZ mice

To test the direct myelinating effect of ACKR3 antagonism, ACT-1004-1239 was evaluated on the spontaneous remyelination process occurring after cuprizone removal. In the first study, the effect of ACT-1004-1239 treatment on myelination was evaluated head-to-head with the antihistaminergic clemastine fumarate. Clemastine, a known accelerator of oligodendrocyte maturation and myelination both in vitro and in vivo, served as a positive comparator [28]. Oral treatments were only initiated after 5 weeks of cuprizone exposure (when demyelination is described to culminate [29]) and continued for 2 to 3 weeks (1 and 2 weeks after cuprizone cessation, respectively) (Fig. 7A). Cuprizone given as food admix for 6 weeks induced significant demyelination in the corpus callosum. This demyelination persisted even one or two weeks after discontinuation of cuprizone, as shown by a decrease in the intensity of the LFB^+^ area, reaching 55% and 48% of demyelination, respectively, compared to mice fed with control food (Fig. 7B). In accordance with literature, treatment with clemastine significantly increased the spontaneous remyelination occurring in the corpus callosum by 32%, 2 weeks after cuprizone removal (*p* = 0.043 versus vehicle-treated mice). While clemastine only started to have a significant positive effect on myelination after 3 weeks of treatment, ACT-1004-1239 treatment led to faster and significantly accelerated spontaneous remyelination by 43% already 1 week after cuprizone removal as compared to vehicle-treated/cuprizone exposed mice (*p* = 0.007) (Fig. 7B-C). In contrast to clemastine, the positive impact on myelination observed with ACKR3 antagonism was not associated with a decrease in cuprizone-induced astrogliosis (Fig. 7D) and microgliosis (Fig. 7E), suggesting that these two drugs induce remyelination via different mechanisms of action.

To exclude any potential impact of ACT-1004-1239 on cuprizone-induced toxic effects, the efficacy of ACT-1004-1239 on remyelination was further investigated in a second independent experiment where treatment was initiated only after cessation of cuprizone exposure (Fig. 7F). Mice exposed to cuprizone for 5 weeks followed by 2 weeks of normal chow demonstrated a severe demyelination in the corpus callosum (84% of demyelination) compared to mice fed with control food. Treatment with ACT-1004-1239 at 100 mg/kg, b.i.d., p.o. for 2 weeks, starting at the time of cuprizone removal (study week 5), significantly accelerated remyelination within the corpus callosum, increasing the mean myelinated area by 174% compared with vehicle-treated mice (43.46 ± 6.86% vs. 15.85 ± 5.03% respectively, *p* = 0.009) (Fig. 7G).

### Efficacy of ACT-1004-1239 on Remyelination is Associated with a CXCL12 Increase in Brain Tissue

**Fig. 8:**
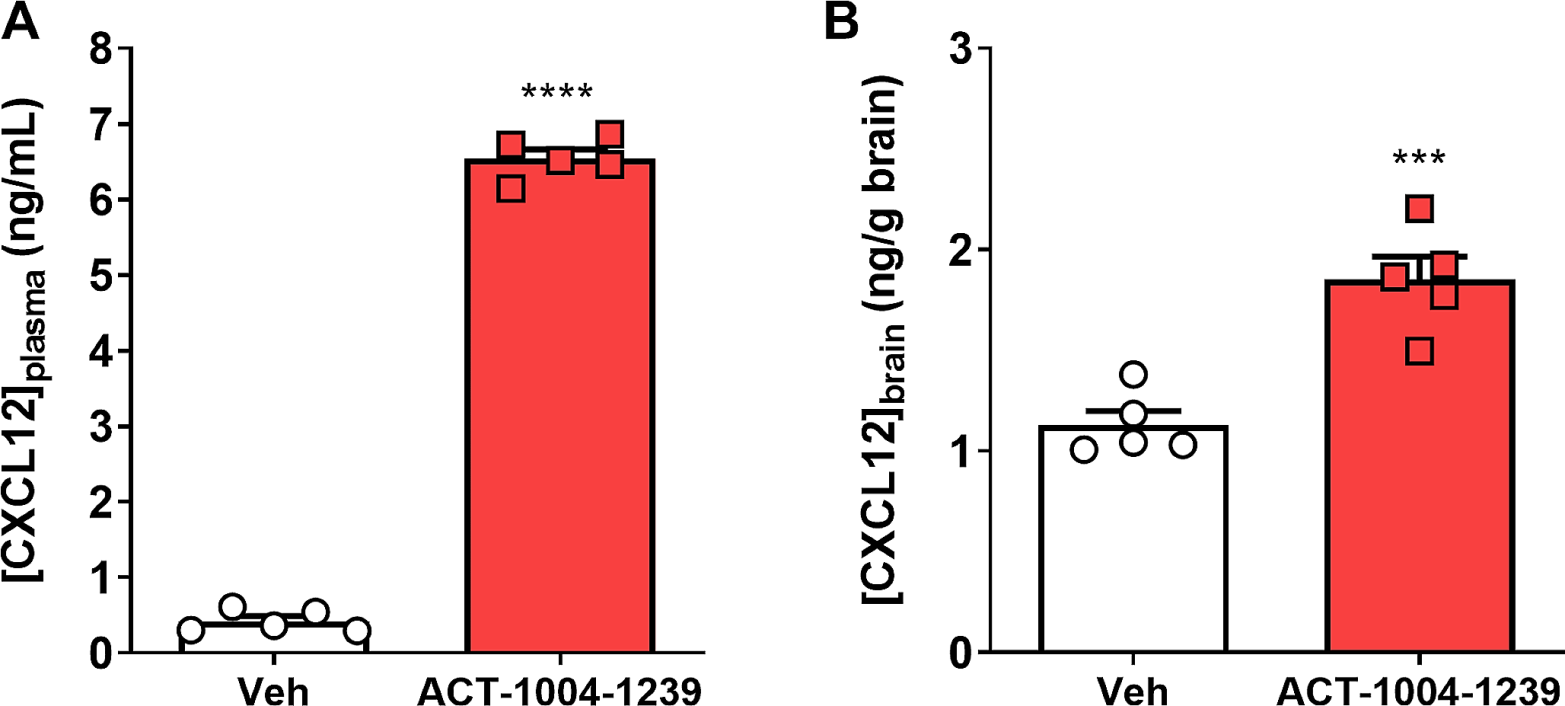
ACT-1004-1239 increases the levels of CXCL12 in both plasma and brain tissue. Healthy female C57BL/6 mice were orally treated with vehicle or ACT-1004-1239 (100 mg/kg), twice daily for 3 days, for a total of 5 doses. Blood and brain samples were collected 1 h after the last oral administration and analyzed for CXCL12 concentrations. ACT-1004-1239 significantly increased (**A**) plasma and (**B**) brain tissue CXCL12 concentrations. Results are expressed as mean + SEM, *n* = 5/group. ***p < 0.001, ****p < 0.0001 using t-test versus vehicle-treated mice

Increase of CXCL12 in the CNS has been proposed to be one of the mechanisms by which ACKR3 antagonism could induce OPC maturation and subsequent myelination [6, 8]. To assess ACT-1004-1239 pharmacokinetic in the brain tissue versus plasma, C57BL/6 mice were treated orally with ACT-1004-1239 at 100 mg/kg and samples were collected 0.5, 1 h, 2.5 h and 4 h after treatment. Concentrations of ACT-1004-1239 reached a maximum (C_max_) in plasma and brain tissue 0.5 h after the last oral administration with a total C_max_ of 4580 ng/mL and 276 ng/g, respectively (Supplementary Table 1). Approximately 6% of drug levels were measured in brain homogenates, indicating limited brain penetration (Supplementary Table 1). To assess whether this CNS exposure was pharmacologically active, C57BL6 mice were treated orally with vehicle or ACT-1004-1239 at 100 mg/kg, twice daily for 3 days and samples were collected 1 h after the last dose to measure CXCL12 concentrations. Treatment with ACT-1004-1239 induced an elevation of CXCL12 in both plasma and brain tissue as compared to vehicle-treated mice (*p* < 0.0001 and *p* = 0.0007, respectively versus vehicle-treated mice) (Fig. 8A-B). This demonstrates effective brain target engagement in vivo at a dose leading to remyelination.

## Discussion

The chronic disability and disease progression in MS are attributed to demyelination and axonal degeneration, for which an effective treatment is lacking [30]. Immunomodulatory strategies have proven successful in reducing the number of relapses and influencing the onset of progressive disability; however, they fail to completely prevent progression [31, 32]. Remyelination is an inefficient process in the inflammatory milieu of MS lesions [30, 33]. Hence, an ideal therapeutic strategy would have the potential to both dampen CNS inflammation and enhance the repair of the myelin sheath. Recently, ACT-1004-1239, a first-in-class, potent, selective, and orally available ACKR3 antagonist, has demonstrated such a dual mechanism of action in EAE mice when administered before disease onset [6]. In the present study, the administration of ACT-1004-1239, initiated on already established disease, demonstrated a dose-dependent reduction in disease severity in the PLP-induced EAE model. In addition, ACT-1004-1239 showed acceleration of remyelination in the cuprizone-induced demyelination model. These findings reinforce the proposed dual mechanism of action of ACT-1004-1239 demonstrating immunomodulatory and remyelinating capabilities.

MS displays a notable gender bias in humans, with a higher prevalence among women who predominantly experience relapsing-remitting forms of the disease. Similarly, the EAE model in the SJL mouse strain, in contrast to the C57BL/6 mouse strain, expresses both chemokine ligands of ACKR3, and demonstrates a comparable sexual dimorphism. Female mice in this model display a relapsing-remitting clinical course [34], providing a compelling rationale for using this particular model. ACT-1004-1239 treatment, initiated at EAE onset, dose-dependently reduced the overall clinical severity of mouse EAE. Amelioration of EAE with ACT-1004-1239 was highlighted by improved histological outcomes in the spinal cord. Efficacy on clinical score was associated with an increase of plasma CXCL11 and CXCL12 levels in EAE mice, confirming the blockade of ACKR3 scavenging activity. Moreover, both 100 and 150 mg/kg doses, given orally twice daily in EAE mice, demonstrated sufficient plasma drug concentration at trough to block more than 90% of the mouse ACKR3 molecules [17]. Consistent with prior data [15], the plasma concentration of CXCL12 approached a plateau of elevation at doses equal or exceeding 100 mg/kg, twice daily. This phenomenon was associated with the observed maximum efficacy in reducing the overall extent of EAE disease. Interestingly, unlike most of the available disease-modifying therapies that commonly result in lymphopenia [24], ACT-1004-1239, when administered at efficacious doses, led to a slight increase in both blood B and T lymphocytes. Specifically, there was an elevation in blood effector/effector memory and central memory T cells numbers, suggesting a potential inhibition of migration of these cells into the CNS during EAE. In line with this observation, elevation of both CXCL11 and CXCL12 plasma levels has been previously associated with reduced leukocyte migration to the site of inflammation [6, 18, 35]. Consistent with these findings, preventive administration of ACT-1004-1239 to PLP-immunized mice prevented CNS infiltration of immune cells prior disease onset. Literature data suggest that increasing systemic CXCL11 and CXCL12 concentrations might also shift the polarization of T cells towards a regulatory phenotype [36, 37]. However, in the present study, preventive administration of ACT-1004-1239 did not change the polarization of activated immune cells in lymph nodes. In addition, the reduction of CNS immune cells infiltration could not be explained by a direct effect of ACT-1004-1239 on CXCL12-induced leukocyte migration in vitro.

Taken together, these data strengthen the hypothesis that ACT-1004-1239, through the blockade of ACKR3 scavenging activity, increases CXCL11 and CXCL12 plasma concentrations, thereby disrupting these chemokine concentration gradients, and impeding the directional migration of leukocytes into the CNS during neuroinflammation.

S1PR agonists are known to be highly effective in EAE models [38, 39], reducing both disease incidence and clinical disease score, most likely via inhibition of autoreactive lymphocytes infiltration into the CNS [40]. Therapeutic efficacy observed in EAE models translated to clinical efficacy in phase 3 trials involving patients with RRMS. In addition, siponimod, a S1PR 1, 5 modulator, has demonstrated its effectiveness in reducing 3-month confirmed disability progression and brain atrophy compared with placebo in secondary progressive MS [41]. Therapeutic administration of ACT-1004-1239 or siponimod, given at doses leading to clinically relevant pharmacodynamic effects, namely maximal plasma CXCL12 increase and maximal lymphopenia over 24 h, respectively, demonstrated similar efficacy on EAE clinical scores and relapse rate. The combination of ACT-1004-1239 with siponimod demonstrated superior efficacy over each monotherapy in the EAE model. Specifically, combination therapy reduced overall clinical scores, and relapse rate. When considering the combination of different drugs, it is crucial to minimize the risk of overlapping or increasing toxicity. Sustained lymphopenia is a well-documented pharmacodynamic effect of S1PR modulators, including siponimod [15]. In contrast, ACT-1004-1239 does not cause lymphopenia, and the combination therapy did not result in further blood lymphocyte count reduction as compared to siponimod monotherapy. In addition, the increase in plasma CXCL11 and CXCL12 concentrations induced by ACT-1004-1239 was not affected by the addition of siponimod. Therefore, the observed synergistic effect on EAE severity in the combination group cannot be attributed to the elicitation of a stronger PD effect. Instead, it suggests that both drugs have distinct mechanisms of action that appear to act in a complementary manner, resulting in increased efficacy in EAE.

There is currently no drug intervention for neuroprotection in neuroinflammatory demyelinating and neurodegenerative disorders, mainly due to an incomplete understanding of the mechanisms underlying axonal degeneration. Recent studies propose a key role of the myelin sheath in supporting axon survival by providing both physical and metabolic support. Therefore, enhancing remyelination is recognized as a promising approach to promote neuroprotection, hence restoring nerve conduction and preventing disability progression [30]. EAE is recognized as a model of neuroinflammation-induced demyelination, characteristic of RRMS. However, the highly inflammatory response and the random distribution of demyelinated lesions make it challenging to assess CNS intrinsic effects leading to disease progression and remyelination [42]. The cuprizone-induced model is primarily used to study the mechanisms of primary demyelination independent of an inflammatory insult and subsequent remyelination.

In previous studies, ACT-1004-1239 co-administrated with cuprizone was shown to reduce demyelination in the corpus callosum of mice by enhancing maturation of OPCs into myelinating oligodendrocytes trough the CXCR4/CXCL12 axis [6]. In the present study, the effect of ACKR3 antagonism was assessed on the spontaneous remyelination process occurring after the injury, after cessation of cuprizone exposure. Treatment with ACT-1004-1239, initiated after 5 weeks of cuprizone exposure, when demyelination culminates, significantly increased remyelination in the corpus callosum. In line with previous results obtained with a functional ACKR3 antagonist in the cuprizone model [8], promyelinating effects of ACT-1004-1239 were associated with an increase in CXCL12 levels, both in the plasma and CNS, demonstrating target engagement in both compartments. Importantly, this remyelination enhancement, unlike the positive control clemastine, did not correlate with a reduction in cuprizone-induced reactive astrogliosis nor microgliosis. This suggests that while both drugs have been shown to promote OPC maturation, they may also act on distinct aspects of the cuprizone-induced demyelination pathology. Recent research has revealed that clemastine boosts myelin recovery without relying on contributions from surviving oligodendrocytes [33]. In the present study, while clemastine required 3 weeks of treatment to induce any visible remyelination, ACT-1004-1239 achieved enhanced remyelination already after 2 weeks of treatment, suggesting a faster onset of action. Consequently, it may be worthwhile to investigate whether ACT-1004-1239 influences the surviving oligodendrocytes on top of inducing OPC maturation [43].

## Conclusions

Our results demonstrate that therapeutic administration of ACT-1004-1239, by blocking ACKR3 scavenging activity, increases plasma levels of both CXCL11 and CXCL12 as well as CXCL12 in the brain. This increase in chemokines was associated with reduced CNS immune cell infiltrates and enhanced myelin repair, respectively, resulting in significantly decreased pathology in two preclinical models of MS. Thus, ACKR3 antagonism holds promises to enhance demyelinated lesions repair by addressing two pathogenic mechanisms driving progression of MS, specifically targeting neuroinflammation and demyelination.

### Electronic Supplementary Material

Below is the link to the electronic supplementary material.

Supplementary Material 1

## Data Availability

The data that support the findings of this study are available in the published article and its supplementary files. Other materials are available upon request to the corresponding authors.

## Abbreviations

Bid: Twice Daily
CFA: Complete Freund’s Adjuvant
Cmax: Maximal observed concentration
CNS: Central nervous system
CPZ: Cuprizone
CV-LFB: Cresyl-Violet and Luxol Fast Blue
EAE: Experimental Autoimmune Encephalomyelitis
IFA: Incomplete Freund’s Adjuvant
i.p.: intraperitoneal
MdCs: Monocyte-derived Cells
MS: Multiple sclerosis
NK: Natural Killer
OPC: Oligodendrocyte Precursor Cell
PBS: Phosphate Buffer Saline
pDC: Plasmacytoid Dendritic Cell
PLP: Proteolipid Protein
RR: Relapsing-Remitting
s.c.: subcutaneous
TSA: Tyramide Signal Amplification
