# Supplementary material for: ACKR3 Antagonism Enhances the Repair of Demyelinated Lesions Through Both Immunomodulatory and Remyelinating Effects
Source: Neurochem Res. 2024 May 31;49(8):2087–104. doi: 10.1007/s11064-024-04173-1 (PMC11233362; doi:10.1007/s11064-024-04173-1)
Supplement: Supplementary file 1 — Supplementary Material 1 [file 11064_2024_4173_MOESM1_ESM.docx]

# Supplementary Figures

Supplementary Fig. 1: Therapeutic administration of ACT-1004-1239 dose-dependently reduces clinical severity in the PLP-induced EAE model.

EAE was induced by immunization of female SJL mice with PLP_139-151_/CFA on day 0, and administration of pertussis toxin on days 0 and 2. Control mice were injected with PBS/IFA and used as benchmark control (n=8-9). Vehicle or ACT-1004-1239 (10, 100, or 150 mg/kg) was given orally (p.o.), twice daily (b.i.d.), starting at disease onset for each mouse (therapeutic mode). Mice were assigned to a treatment group at EAE onset, in a blinded manner. (A) Treatment groups were randomized for similar body weight, clinical score at EAE onset, and day of onset. (B) Mean clinical score of EAE mice after treatment initiation, starting at onset of EAE for each mouse. Results are expressed as mean + SEM (n = 14–16 per group). *p < 0.05, **<0.01 vs vehicle-treated EAE mice using two-ways ANOVA followed by uncorrected Fisher’s multiple comparisons test. (C) Mean body weight of EAE mice after treatment initiation. Results are expressed as mean + SEM (n = 14–16 per group). *p < 0.05, vs vehicle-treated EAE mice using mixed-effects followed by uncorrected Fisher’s multiple comparisons test.

Spinal cord samples from control mice and EAE mice treated with vehicle or ACT-1004-1239 at 100 mg/kg, b.i.d., were collected at sacrifice, after 31 to 33 days of treatment for each mouse and embedded in paraffin wax. Sections of 4µm thickness from seven regions of the spinal cord were stained with the primary rabbit anti-CD3 (Abcam) then with a secondary anti-rabbit IgG (Jackson) and revealed with TSA plus Cy3.5. (D) Representative immunohistochemistry images of the spinal cord cervical section of mice. White scale: 80µm (E) Positive CD3 area was quantified on each of the seven spinal cord sections, normalized to the number of nuclei and averaged using OrbitImage analysis software. Results are expressed as mean + SEM, n=9-16 per group. ****p<0.0001 using Kruskall-Wallis test followed with Uncorrected Dunn’s multiple comparisons test vs Veh-treated EAE mice. (F) Correlation between clinical scores assigned to each mouse on the day of sacrifice and average histopathological scores determined by a certified pathologist based on spinal cord sections stained with cresyl violet-luxol fast blue. ****p<0.0001 using spearman correlation test. (G) Correlation between clinical scores assigned to each mouse on the day of sacrifice and the average % of CD3^+^ area quantified in the spinal cord sections from EAE mice. ****p<0.0001 using spearman correlation test.

Supplementary Figure 2: CXCL12 plasma concentrations at trough correlate with blood lymphocyte counts in EAE mice.

EAE was induced by immunization of female SJL mice with PLP_139-151_/CFA on day 0, and the administration of pertussis toxin on days 0 and 2. Vehicle or ACT-1004-1239 (10, 100, or 150 mg/kg) was given orally, twice daily, starting at disease onset for each mouse (therapeutic mode). Blood samples were collected at sacrifice, after 31 to 33 days of treatment for each mouse, 14h after the last oral gavage, at trough. Blood cell subpopulations were analyzed by flow cytometry and plasma samples were analyzed for CXCL11 and CXCL12 plasma concentrations. Correlation between CXCL12 plasma concentrations and T cell counts (A) or B cell counts (B) at trough. ***p<0.001, ***p<0.0001 using Pearson correlation test.

Supplementary Figure 3: CXCL11 does not induce in vitro chemotaxis from freshly isolated splenocytes from PLP-induced EAE mice

EAE was induced by immunization of female SJL mice with PLP_139-151_/CFA on day 0, and administration of pertussis toxin on days 0 and 2. (A) Spleens were collected, processed, and assessed in an in vitro CXCL11 migration assay. Results are expressed as mean + SD, (triplicates/condition). (B) CXCR4 antagonism (CXCR4 ant) with AMD3100, ACKR3, or CXCR3 antagonism (ACT-1004-1239 or AMG287, respectively) did not affect splenocytes migration. Results are expressed as mean + SD. (Each condition was performed in triplicates which were averaged for each mouse, n=3 mice).

Supplementary Figure 4: Combination therapy does not affect the pharmacodynamic response of each monotherapy nor drug exposure at trough.

EAE was induced by immunization of female SJL mice with PLP_139-151_/CFA on day 0, and administration of pertussis toxin on days 0 and 2. Vehicle, ACT-1004-1239 (150 mg/kg, twice daily), siponimod (0.1 mg/kg, once daily) or the combination of ACT-1004-1239 with siponimod was given orally, starting at disease onset for each mouse (therapeutic mode). One day before the end of the study, blood was collected via tail vein puncture from representative mice of vehicle, siponimod, and combination-treatment groups (n=4/group) to assess T cell (A) and B cell (B) counts by flow cytometry, and assess plasma concentration of siponimod (C), 24 hours after the last administration of siponimod (trough). Results are expressed as mean + SEM. ***p<0.001, ****p<0.0001 using one way ANOVA followed by Dunnett’s multiple comparison test. ns: non-significant using t-test. (D-F) At the end of the study, blood was collected via vena cava puncture and plasma samples were prepared at sacrifice, after at least 32 days of treatment for each mouse, 14 hours after last dosing (at trough for ACT-1004-1239 treatment). (D) CXCL11 and (E) CXCL12 plasma concentrations at trough. Results are expressed as mean + SEM (n=15-17 group). ***p<0.001, ****p<0.0001 versus vehicle-treated EAE mice, using one-way ANOVA followed by Dunnett’s multiple comparisons test. (F) ACT-1004-1239 plasma concentrations measured at trough, expressed as mean + SEM (n=15-16 per group). The dotted line represents the concentration inhibiting 90% of ACKR3 molecules (mouse IC_90_) based on an in vitro β-arrestin recruitment assay. ns: non-significant using t-test. Low limit of quantification is 4.57 ng/mL. In addition, (G) T cell and B cell (H) counts were quantified by flow cytometry at the end of study, 14 hours after the last oral administration. Results are expressed as mean + SEM (n=15-17 group). ***p<0.001, ****p<0.0001, using one-way ANOVA followed by Tukey’s multiple comparisons test.

**Supplementary Table 1: Pharmacokinetic parameters of oral ACT-1004-1239 at 100 mg/kg in plasma and brain tissue in C57BL/6 mice.**

|  | **AUC_0-last_ (ng*h/mL or g)** | **C_max_ (ng/mL or g)** | **T_max_ (h)** |
| --- | --- | --- | --- |
| **Plasma** | 10100 | 4580 | 0.5 |
| **Brain** | 673 | 276 | 0.5 |

ACT-1004-1239 (100 mg/kg) was given orally to C57BL/6 mice. Blood was collected 0.5, 1, 2.25 and 4h after the oral administration. Observed PK parameters are expressed as geometric mean (n=3 mice/time-point). Low limit of quantification of 1.5 ng/mL. C_max_: maximal observed concentration; T_max_: time of maximal observed concentration; AUC_0-last_: area under the plasma or brain concentration vs time curve up to the last measurable concentration, calculated by the log-linear trapezoidal rule. Parameters were estimated using the Phoenix 6.4 software package (Pharsight Corporation, Cary, NC, USA) using non-compartmental analysis and the linear up/log trapezoidal down calculation method without weighting.
